# Geographic Disparities in Online Searches for Psoriasis Biologics in the United States: Google Trends Analysis

**DOI:** 10.2196/56406

**Published:** 2024-07-31

**Authors:** Annie Chang, Ross O'Hagan, Jade N Young, Nancy Wei, Nicholas Gulati

**Affiliations:** 1 Department of Dermatology Icahn School of Medicine at Mount Sinai New York, NY United States

**Keywords:** psoriasis, biologics, health disparities, Google Trends, online search, web-based search, USA, United States, Google, awareness, skin, patient awareness, psoriasis treatment, US, psoriasis medication, patient education

## Introduction

Twelve biologics targeting cytokines TNF-α (tumor necrosis factor alpha), IL-12/23 (interleukin 12/23), IL-17 (interleukin 17), and IL-23 (interleukin 23) have been approved for the treatment of moderate to severe psoriasis in the United States, including most recently bimekizumab in October 2023 [1,2]. In this study, we used publicly available Google Trends data to monitor search volumes for psoriasis biologics, a methodology that has been used in prior studies [3,4].

## Methods

All 12 US Food and Drug Administration (FDA)–approved psoriasis biologics were included in our analysis. We examined temporal search volume data from each biologic’s approval date for plaque psoriasis until November 1, 2023, and geographic search volume data over the past 12 months (November 2022 to November 2023). Search volume was indicated by a relative search volume (RSV) index, scaling from 0 (no searches for that medication) to 100 (peak search volume for that medication). This index is calibrated in relation to each state’s total search volume within the United States and specified time range. Trend analysis was conducted using the Mann-Kendall test in R software (version 4.3.1; The R Foundation).

## Results

**Figure 1:**
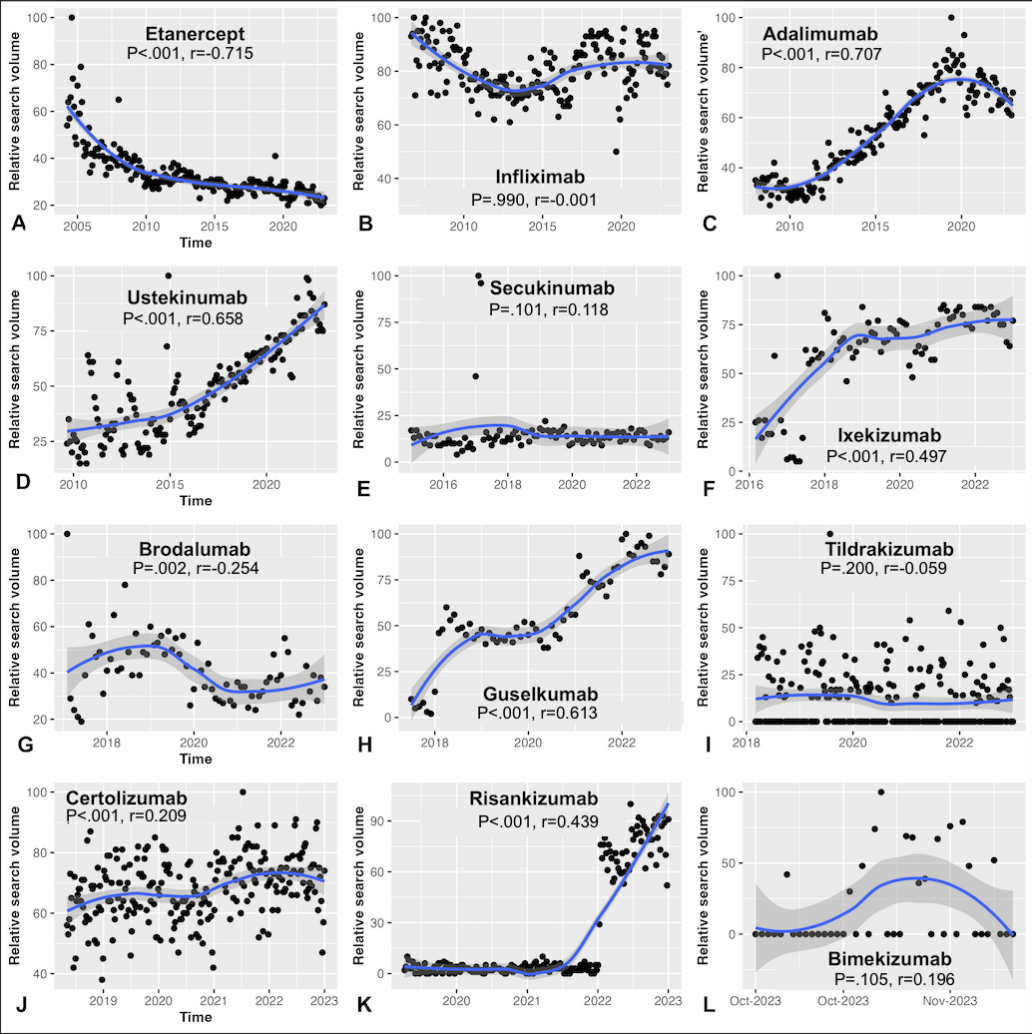
Temporal trends in relative search volume for psoriasis biologics after approval by the US Food and Drug Administration.

**Figure 2:**
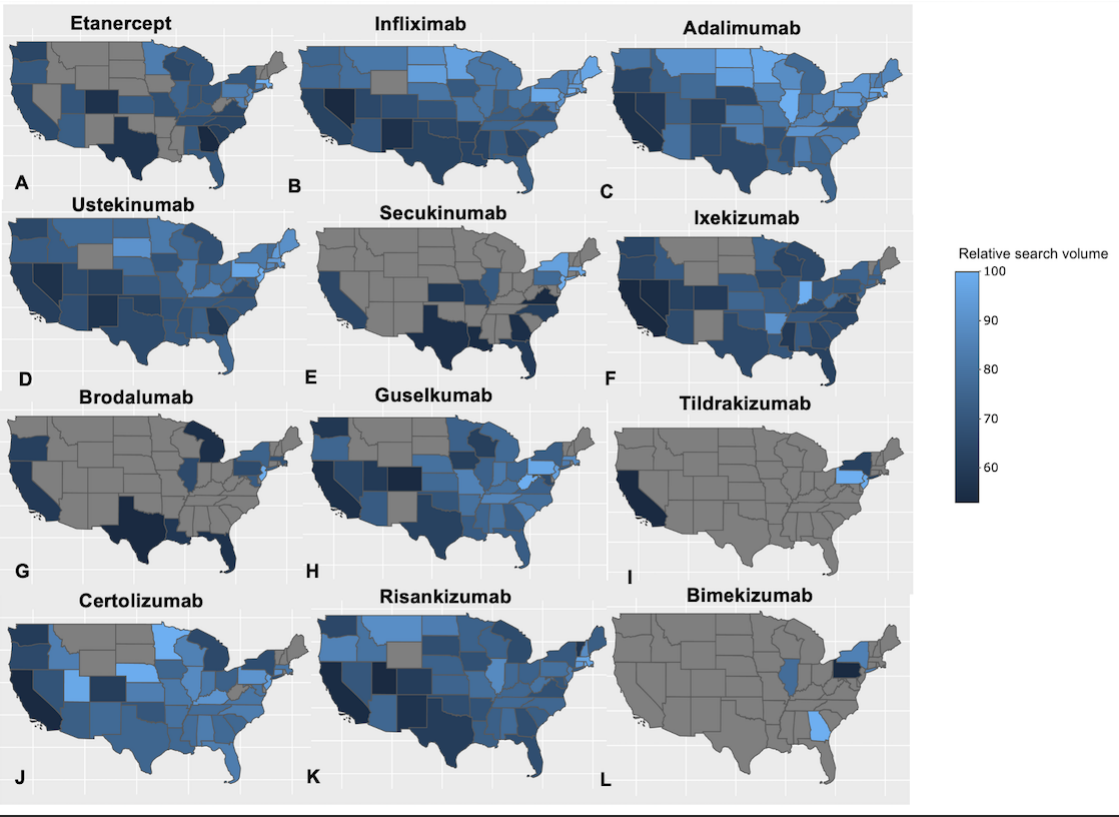
Geographic distribution of relative search volume for psoriasis biologics in the past 12 months (November 2022 to November 2023) across the United States. The relative search volumes are based on each state’s total search volume within the designated geography and time frame. The color scale ranges from 0 (no searches) to 100 (peak search volume), with the gray in the maps representing no data available.

Overall, our analysis of search trends over time revealed increasing public interest in most psoriasis biologics (Figure 1) based on RSVs. Rising trends in RSVs since FDA approval were observed for adalimumab, ustekinumab, ixekizumab, guselkumab, certolizumab, and risankizumab. Declining RSVs were observed for etanercept and brodalumab, while search volumes were generally stable for infliximab, secukinumab, tildrakizumab, and bimekizumab. For detailed RSV ranges, please refer to Multimedia Appendix 1. It is important to note that these RSVs represent the search interest in each medication relative to the total search volume within the specified region and time period rather than absolute search volumes. Therefore, while risankizumab has shown increased relative popularity, this does not necessarily imply that its absolute search volume surpasses that of older medications like infliximab. Over the past year, geographic analysis revealed heterogeneous public interest patterns across biologics in the United States (Figure 2). Coastal states, particularly California and the Eastern seaboard, had higher RSVs, whereas Midwestern states had the lowest. In many areas of the United States, IL-17 and IL-23 inhibitors remain among the lesser-searched psoriasis treatments. Notably, risankizumab demonstrated a rapid rise in nationwide search volume from mid-2021 (*P*<.001).

## Discussion

Our results highlight consistently low Google search volumes for many standard-of-care psoriasis biologics across the United States, indicating disparities in patient awareness regarding these treatments. These findings add to previous studies showing regional disparities in psoriasis medication use and treatment outcomes in the United States, including research indicating that Southern states had the highest proportion of patients receiving psoriasis biologics per year based on the 1996-2015 Medicare Expenditure Panel Survey [5,6]. Furthermore, while risankizumab has shown increased popularity, it does not necessarily correlate with clinical superiority. A recent meta-analysis of psoriasis randomized controlled trials found no significant difference in efficacy, defined as the proportion of patients who achieved Psoriasis Area and Severity Index (PASI) 90, 8 to 24 weeks following treatment onset, between risankizumab, infliximab, bimekizumab, and ixekizumab [7].

To our knowledge, this study is the first to examine disparities in patient awareness of psoriasis medications both geographically and longitudinally. While an RSV analysis does not provide information on absolute search volumes, it offers valuable insights into the relative popularity and public interest in different psoriasis medications across regions and over time. By analyzing relative trends for each medication, we can identify medications that are gaining or declining in awareness, which can inform efforts to improve patient education.

A limitation of our study is the nonspecific nature of search queries, which may not relate exclusively to psoriasis (eg, “etanercept” searches could include other indications). This likely underestimates the disparity in searches between TNF-α agents and newer biologics, suggesting that the observed decline in etanercept searches could be more pronounced than indicated. Incorporating per capita search data in future research could improve our understanding by providing a normalized metric that reflects search interest adjusted for population size. This study serves as an initial investigation into the utility of online search trends as a proxy for public awareness of psoriasis biologics, underscoring the need for comprehensive patient education on the wide array of available psoriasis treatments.

## Appendix

Multimedia Appendix 1 Minimum and maximum relative search volumes for each biologic.

## Abbreviations

FDA: Food and Drug Administration
IL-12/23: interleukin 12/23
IL-17: interleukin 17
IL-23: interleukin 23
PASI: Psoriasis Area and Severity Index
RSV: relative search volume
TNF-α: tumor necrosis factor alpha
