# Supplementary material for: Geographic Disparities in Online Searches for Psoriasis Biologics in the United States: Google Trends Analysis
Source: JMIR Dermatol. 2024 Jul 31;7:e56406. doi: 10.2196/56406 (PMC11325122; doi:10.2196/56406)
Supplement: Multimedia Appendix 1 [file derma_v7i1e56406_app1.docx]

**Multimedia Appendix 1**. Minimum and maximum relative search volumes (RSV) for each biologic.

| Biologic | Minimum RSV | Maximum RSV |
| --- | --- | --- |
| Etanercept | 20 | 100 |
| Infliximab | 30 | 100 |
| Adalimumab | 25 | 100 |
| Ustekinumab | 15 | 100 |
| Secukinumab | 4 | 100 |
| Ixekizumab | 5 | 100 |
| Brodalumab | 19 | 100 |
| Guselkumab | 2 | 100 |
| Tildrakizumab | 0 | 100 |
| Certolizumab | 38 | 100 |
| Risankizumab | 0 | 100 |
| Bimekizumab | 0 | 100 |
